# Clinical Manifestations, Diagnosis, and Surgery of Inferior Oblique Muscle Ectopia

**DOI:** 10.1155/2020/3039180

**Published:** 2020-04-21

**Authors:** Zhipeng Xue, Xiaoshan Min, Jieyue Wang, Ying Zhu, Sujun He, Kangcheng Liu, Yi Ding

**Affiliations:** ^1^Department of Ophthalmology, Xiangya Hospital Central South University, Changsha 410000, Hunan, China; ^2^Changsha Jiqiang Eye Hospital, Changsha 410000, Hunan, China

## Abstract

**Objectives:**

To summarize the clinical manifestations, diagnosis, treatment, and prognosis for inferior oblique muscle ectopia (IO-E). *Subjects and Methods*. Patients diagnosed with IO-E during strabismus surgery from March 2017 to September 2018 were included in this retrospective, cross-sectional study. All patients received preoperative Krimsky test, synoptophore, cycloplegia refraction, fundus torsion, and other strabismus-related specific tests. The anatomic variations of IO-E were always discovered during surgical procedure. Postoperative eye position and binocular visual function (BVF) were all reviewed in early days after operation.

**Results:**

A total of 7 patients were enrolled in this study with an average age of 6.4 ± 3.8 yrs. They all presented with significant exotropia and unilateral (or bilateral) overelevation in adduction (OEA). No compensatory head position was detected. Some of them had vertical deviation, V pattern, or excyclotropia, which were indicated by fundus torsion. Monocular or binocular IO-E was distinguished during the surgery, and it could be classified into two types according to its anatomic features. In surgery, the ectopic IO muscle bundle was restored, and different IO weakening methods were employed. Meanwhile, the horizontal deviation was also corrected according to the preoperative examination. Eyes of all patients were properly aligned in the primary position after surgery. Varying degrees of BVF appeared in 3 cases.

**Conclusions:**

IO-E is a rare congenital dysplasia variation of the extraocular muscle, which could appear as inferior oblique overaction. It is difficult to diagnose before surgery, and weakening the overactive ectopic inferior oblique was required for better prognosis if this condition was confirmed during surgery.

## 1. Introduction

Anatomical abnormalities of the inferior oblique (IO) are quite rare in clinical practice; however, the accurate diagnosis and proper management could determine the prognosis of surgery. There were only a few case reports about inferior oblique ectopia (IO-E) so far. Wang Xiaojun reported a case in which the insertion of the IO tendon into the sclera displaced retroposed [1]. In the three cases of IO-E reported by Lin in 2012, in one case, the IO tendon was ante displacement, while in the other two cases, the IO bundle was split and fused with the lateral rectus [2]. Huiqin and Guan found one case in which the IO attached to the sclera backwardly, and the muscle structure was abnormal [3]. Qureshi and Watson reported a case of absence of the IO [4]. But, none of them discussed the possible impacts of IO-E on ocular movement, strabismus surgery design, and prognosis. In the present study, we conducted a retrospective analysis of the IO-E cases we encountered in the past two years in order to shed light on the diagnosis and treatment for this rare disease.

## 2. Subjects and Methods

The study adhered to the tenets of the Declaration of Helsinki and was approved by the Institutional Review Board of Xiangya Hospital, Central South University. In this retrospective study, charts of patients with strabismus who underwent strabismus surgery by one skilled pediatric ophthalmologist (XSM) between March 2017 and September 2018 were reviewed.

Seven cases of IO-E diagnosed under direct observation during surgery were included in our study. All patients underwent detailed systemic examination and ophthalmologic examination before surgery to rule out surgical contraindications. Preoperative strabismus examinations included the Krimsky test (conducted in 33 cm), ocular movement, compensatory head posture observation, synoptophore, cycloplegia refraction, and fundus torsion. The Krimsky test, synoptophore, and fundus torsion were also performed in several days (1–3 days) after surgery. Orbital MRI or other imaging examinations were not performed as a routine regimen for strabismus patients.

All strabismus surgeries were conducted under general anesthesia. The design and implementation of the surgical plan were carried out by the same surgeon. Surgical plans were based on the patients' preoperative strabismus examination: horizontal rectus resection/recession was applied to eliminate the obvious exotropia, and IO weakening procedures were combined to deal with overelevation in adduction (OEA): if OEA>1+, IO transposition was performed; otherwise, we chose IO tenotomy. When IO-E was determined during operation, predetermined surgical procedures were still performed after operation on the ectopic IO muscle.

## 3. Results

Seven IO-E patients in our study included 2 boys and 5 girls. They all came to our hospital because of exodeviation. The course of disease ranged from 1 to 9 yrs. The average age at the time of surgery was 6.4 ± 3.8 yrs (range: 2.9 yrs. to 14 yrs.). No patients had history of premature birth or dystocia. The growth and development were similar to peers. All patients denied the history of ocular trauma and other ocular operations.

Results of preoperative strabismus examinations were summarized in Table 1. The average exodeviation was −55 ± 12.9 PD (range −30∼−70 PD), all patients showed OEA, and none had compensatory head position, some with V-pattern exotropia or excyclotropia.

Eight eyes of the seven patients were diagnosed with IO-E. The variation of the ectopic IO muscle could be divided into two parts: anterior and posterior bundle, and usually, the path of the anterior bundle was variable. Based on the abnormal path and sclera insertion of the anterior bundle, IO-E could be classified into the following two types.

**Figure 1:**
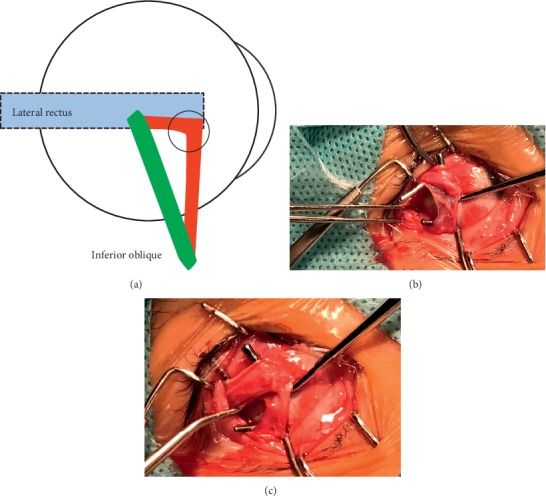
Type-*α* IO-E: the anterior IO bundle (in red) loosely adhered with the lower part of lateral rectus insertion (purple circle) and then turned backwardly, joined the posterior IO bundle (in green), and ended at its conventional insertion.

Type-*α*: the anterior bundle of the IO muscle, encased with the transparent fascia membrane, in some cases fan-shaped, courses posteriorly after loosely adhering to the lateral rectus insertion and inserts in the conventional position together with the posterior bundle of the IO muscle (see Figure 1). Four cases of type-*α* IO-E were included in our study. All presented with vertical deviation, and the higher eyes were exactly the eye with IO-E. Two patients had V-pattern exotropia and excyclotropia.

**Figure 2:**
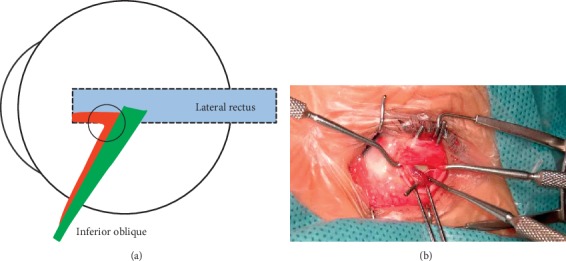
Type-*β* IO-E: all IO fibers passed normally to their conventional insertion, while the posterior bundle (in green) fused tightly, and the anterior bundle (in red) loosely connected with the IO insertion (purple circle) and went forward under the lateral rectus and fused with lateral rectus insertion.

Type-*β*: the IO muscle passes normally to its conventional insertion, and the posterior bundle fuses with the sclera tightly, while the anterior bundle loosely connects with IO insertion and then turns anteriorly, passes under the lateral rectus, and fuses with lateral rectus insertion (see Figure 2). In this study, there were 3 cases (4 eyes) with type-*β* IO-E: one of them had bilateral IO-E, one patient had a V-pattern exotropia, and another one had a bilateral excyclotropia. None of them had vertical strabismus.

Krimsky test, synoptophore, and fundus torsion were conducted in one to three days after surgery; three cases achieved different levels of binocular single vision function. Two children could not cooperate with the examination because of their young age (see Table 2).

## 4. Discussion

Clinical manifestations and treatments of the IO muscle dysfunction have been reported frequently, but anatomic abnormalities of the IO muscle are rarely reported. This study retrospectively analyzed the clinical manifestations, details of IO muscle anatomic abnormality, and surgical outcomes of 7 patients diagnosed with IO-E during strabismus surgery and detected two types of IO-E, aiming to provide some reference for the design of surgical plans for strabismus patients with IO-E in the future.

In the seven IO-E patients, the ocular deviation was mostly observed around the age of 2 yrs. Their medical history was carefully reviewed. All had no history of birth trauma, ocular trauma, or eye-related diseases. Therefore, the IO-E might be a variation during the development of the extraocular muscles. Embryonic development of the extraocular muscles is correlated with the development of the eyeball. Studies have shown that the extraocular muscle and its tendons originate from the superior or inferior mesenchymal complex, while the lateral rectus muscle originates from both; the IO is derived from the inferior mesenchymal complex [5, 6]. The compact spatial location of the IO and lateral rectus muscle origination may be the cause of their abnormal adhesion. The tendons of rectus muscles reach their adult sclera insertion until the ages of 18 months to 2 years [5]. During the development, changes in a variety of regulatory factors including genes, brainstem ocular motor neurons, growth factors, and hormones may lead to abnormalities of extraocular muscles [7].

All the IO-E patients we analyzed showed significant exotropia and OEA, and none of them had a compensatory head posture. Meanwhile, we noticed that there were different features of deviation in two types of IO-E: firstly, all patients with *α*-type IO-E had a positive head-tilt test; secondly, vertical deviation occurred only in patients with *α*-type ectopic, and the higher eye happened to be the eye with ectopic IO. This difference impelled us to reflect on how these two types of IO-E affect ocular position and eye movement.

The IO muscle originates from the fossa outside the opening of the nasolacrimal duct in the maxilla. It passes posteriorly, superiorly, and laterally between the inferior orbital wall and the inferior rectus muscle, inserting in the lower edge of the lateral rectus muscle with a concave arc. The anterior part of insertion is 10 mm far from the lower ending of the lateral rectus muscle, the posterior part of insertion is 4.2 mm far from the optic nerve, and only 2.2 mm away from the fovea of the macula [8]. The contact curve between the IO muscle and the eyeball is 15 mm, which is the longest one of all extraocular muscles, and it might be responsible for IO-E.

Because the eye rotation center is between the origin and insertion of the IO muscle and the IO muscle plane forms an angle of 51° with the visual axis of the eye in the primary position, IO muscle's primary function is extortion; secondary action is elevation and abduction [8]. When the eye adducts, its primary action turns to be elevation.

Significant exotropia was the primary clinical manifestation in these IO-E patients, and all of them showed OEA. Since abduction is the secondary function of the IO muscle, little contribution was made by the IO muscle to exotropia. Therefore, the surgical plan mainly eliminates exotropia by adjusting the power of the lateral and medial rectus.

It is generally believed that OEA is a result of inferior oblique overaction (IOOA): when the eyeball moves inward to the nose, the angle between the visual axis and the IO muscle plane is reduced, which would enhance the elevation of the IO muscle and lead to elevation of the eyeball [9, 10]. IO muscle weakening procedures were designed preoperatively to deal with IOOA. When OEA≤1+, IO myotomy was performed; otherwise, IO transposition was adapted. However, when IO-E was discovered during surgery, which is the proper surgical procedure? First of all, we need to determine the effect of the ectopic IO muscle on IOOA. We believe that these two types of IO-E mentioned above can aggravate the manifestation of IOOA for the following two reasons:

- In the case of adduction, the ectopic anterior IO muscle bundle would approach the posterior muscle bundle and increase the strength of the IO.
- The effective insertion moves forward in type-*α* and type-*β* IO-E, so the angle between IO muscle and visual axis increases, which enhances extortion action of the IO muscle. In fact, patients with IOOA often show excyclotropia and V-pattern exotropia [11, 12]. Kushner et al. found that patients with primary IOOA were usually accompanied by excyclotropia [10, 13]. They believed the enhancement of the primary or secondary action (torsion and elevation) of the oblique muscle would strengthen the third action (abduction) which means while the eyeball elevated, the abduction was enhanced, which explains the V-pattern exotropia in some patients [14]. Some of the 7 patients showed excyclotropia, and 3 patients showed V-pattern exotropia, which indicates IO-E and IOOA occurred concurrently.

Based on the discussion above, the ectopic IO muscle bundle was restored during operation, and the IO muscle was weakened according to the degree of OEA. All patients had no residual vertical deviation, and the visual function recovered well.

By retrospectively reviewing the anatomical structure, clinical features, surgery design, and prognosis of IO-E, our study provided a reference for surgery design when encountered with IO-E. However, this study still has some limitations. Firstly, orbital MRI or other imaging examinations were not acquired before operation to determine whether there was any abnormality of the oblique muscle or orbit. It is also impossible to know whether the commonly used imaging examination can detect IO-E. Therefore, it cannot be excluded whether the nonsurgical eye has IO-E or not. Secondly, the choice of surgical procedures for patients with IO-E was determined by the type of IO-E and the symptoms and signs of the patient by the surgeon during surgery, which was empirical. Thirdly, the number of cases is relatively small; it is impossible to systematically evaluate the efficacy of the relevant procedures for IO-E. According to the follow-up, we can only know that the surgical procedures we used have positive effects on deviation, but it is still unclear whether there are better solutions. However, the ectopic IO muscle bundle dissection and IO muscle weakening were performed simultaneously in all patients; meanwhile, IOOA was eliminated, suggesting that ectopic IO muscle dissection is helpful for relieving IOOA.

## 5. Conclusions

In summary, IO-E is a rare congenital dysplasia, which is supposed to be derived from developmental abnormalities of the extraocular muscles during the embryonic period. There is currently no special preoperative examination to predict whether a patient has IO-E. In strabismus patients with IOOA, if IO-E is determined during surgery, it should be recognized that the ectopic IO muscle bundle can enhance IOOA. After dissecting the ectopic IO muscle bundle, the remaining amount of extraocular muscle surgery can still be calculated according to common strabismus surgery. However, due to the different conditions of IO-E, we need to enroll and summarize more cases to come up with a more effective or specific surgical procedure for strabismus patients with IO-E.

## Figures and Tables

**Table 1:** Demographics and preoperational strabismus examinations.

Case	Age (yrs.)	Sex	Cycloplegia refraction OD/OS	Krimsky test^*∗*^ (PD)	OEA† OD/OS	Pattern and deviation angle (PD)	Fundus torsion OD/OS
1	4.1	M	−0.75DS/−1.25DC*∗*180 +0.50DS/−1.25DC*∗*180	−55 L/R5	0/+1∼+2	—	0
2	2.9	F	+2.00DS +2.00DS	−70 L/R15	+1/+1	35 V−XT‡	Exc 13° Exc 12°
3	8	F	+0.75DS/+0.25DC*∗*80 +0.50DS/+0.50DC*∗*90	−55 R/L10	+1/0	—	0
4	7	F	+1.5DS/+0.75DC*∗*110 +2.00DS/+0.50DC*∗*65	−30 R/L8	+1/+1	25 V−XT	Exc 20° Exc 8°
5	5.3	F	+1.50DS/+0.75DC*∗*90 +1.50DS/+1.00DC*∗*75	−55	+2/0	20 V−XT	0
6	14	M	−1.00DS/−0.75DC*∗*175 +7.00DS	−60	0/+1	—	0
7	3.7	F	+0.75DS/+0.50DC*∗*70 +0.75DS/+0.50DC*∗*75	−65	+1/+1	—	Exc 5° Exc 10°

^*∗*^Krimsky test: in 33 cm; †OEA: overelevation in adduction; ‡V-XT : V-pattern exotropia; Exc: excyclotropia.

**Table 2:** Operation design and binocular single vision restoration.

Case	Type of IO-E and eye	Operation	Binocular vision function	
			Preoperation	Postoperation
1	*α*-OS	IO Tran + LR Rec + MR Res OS	n/a	n/a
2	*α*-OS	IO Ten OD + IO Trans OS + LR Rec + MR Res OU	n/a	n/a
3	*α*-OD	IO Tran + LR Rec + MR Res OD	I	III
4	*α*-OD	IO Tran OD + IO Ten OS + LR Rec OU	Negative	Negative
5	*β*-OD	IO Ten + LR Rec + MR Res OD	Negative	II
6	*β*-OS	LR Rec OU + IO Ten + MR Res OS	Negative	Negative
7	*β*-OU	LR Rec OU + MR Res OD	Negative	II

IO-E: inferior oblique ectopia; LR: lateral rectus; MR: medial rectus; Rec: recession; Res: resection; Ten: tenotomy; Tran: transposition; BVF: binocular vision function; I: simultaneous perception; II: fusion; III: stereopsis; n/a: cannot cooperate.

## Data Availability

In order to protect patient privacy, the clinical information used to support the findings of this study is available from Zhipeng Xue, amazingleo@csu.edu.cn, for researchers who meet the criteria for access to confidential data.
